# Methods for modeling gene-environment interplay using polygenic risk scores

**DOI:** 10.1515/sagmb-2026-0006

**Published:** 2026-09-02

**Authors:** Ziqiao Wang

**Affiliations:** Department of Genome Sciences, University of Virginia, 1335 Lee Street, Charlottesville, VA 22903, USA; and UVA Comprehensive Cancer Center, Charlottesville, VA, USA

**Keywords:** gene-environment interactions, gene-environment correlations, case-parent trio, case-control, case-only, disease risk prediction

## Abstract

Polygenic risk scores (PRS) are increasingly recognized as pivotal tools for quantifying disease risk through the aggregation of multiple genetic variants. As sample sizes in genome-wide association studies (GWAS) continue to expand and PRS become more powerful, they are set to play a key role in translational research and personalized medicine. Understanding the interplay of PRS with environmental factors is critical for interpreting and applying PRS in diverse contexts. This interplay manifests in two forms: PRS-by-environment interaction (PRS × E) and gene-environment correlation (rGE). However, despite the growing application and importance of PRS, there are limited guidelines for performing PRS × E interaction analyses while controlling for rGE, which can lead to inconsistencies across studies and misinterpretation of results. Here we provide a review of different methods for performing PRSxE interaction in various epidemiological study designs, propose recommendations for best-practice, and discuss future challenges.

## 1 Introduction

As the typical sample size for genome-wide association studies (GWAS) continues to increase, polygenic risk scores (PRS), measures of the total genetic burden individuals carry across many genetic variants associated with different traits and diseases, are becoming increasingly predictive of complex traits (Ali et al. 2018; Chatterjee et al. 2016; Khera et al. 2018; Kullo et al. 2022; Lewis and Vassos 2020). PRS are now being integrated into clinical and public health applications for risk prediction, early detection, and precision medicine (Kullo 2025; McHugh et al. 2025). However, realizing the full potential of PRS requires understanding how genetic risk manifests across different environmental contexts. For example, a recent study has shown that a model incorporating PRS × sex interactions improved BMI prediction accuracy by 16 % over a model without this interaction term (Namba et al. 2026). This finding highlights the growing interest in contextualizing PRS with behavioral, sociocultural, and physical environmental factors, particularly for developing joint risk-prediction models that incorporate both genetic and non-genetic factors (Garcia-Closas et al. 2014; Middha Kapoor et al. 2020; Rudolph et al. 2018).

Complex diseases arise from the intricate interplay between the human genome and environmental exposures (Kong et al. 2018). This gene-environment interplay manifests in two primary forms that are critical for interpreting and applying PRS: gene-environment interaction (G × E) and correlation (rGE). G × E refers to the nonadditive or nonmultiplicative effects of genetic and environmental variables on an outcome trait – in other words, how genetic effects vary across environmental contexts. Many complex traits are influenced by genes distributed throughout the genome, each contributing small effects, as described by Fisher’s “infinitesimal model” (Boyle et al. 2017; Fisher 1919). This polygenic architecture has historically limited statistical power to detect interaction at the single nucleotide polymorphism (SNP) level (Ritz et al. 2017), leading modern research to emphasize understanding how the polygenic structure of traits varies across environmental contexts (Herrera-Luis et al. 2024; Miao et al. 2024). PRS-based approaches offer practical advantages for studying interaction, including the ability to analyze curated SNP groups such as pathway-specific PRS (James Gauderman et al. 2025; Westerman et al. 2026), and fitting multiple interaction effects jointly to adjust for correlated interaction effects (Domingue et al. 2020; Wang et al. 2024; Young et al. 2016).

Gene-environment correlation (rGE) can have multiple definitions. In the context of PRS, this includes systematic pleiotropic effects of underlying variants across traits, where the PRS for a disease is correlated with an environmental factor that possesses a heritable component. Another type of rGE can be due to population stratification, when trait differences co-vary with systematic allele frequency differences between sub-populations (Freedman et al. 2004; Leslie et al. 2015; Price et al. 2010). Additionally, rGE can occur across geographical regions, through either active or passive processes (Abdel et al. 2022). Furthermore, rGE occurs within families, i.e., the genomes of close relatives, such as parents and siblings, can affect the proband through their contributions to the proband’s environment (Kong et al. 2018). This is the indirect genetic effects on an individual’s phenotype that occur within families. Assortative mating is another source of rGE (Robinson et al. 2017; Selzam et al. 2019). Behavioral scientists have examined rGE in detail, naming definitions such as passive rGE (within-family indirect effect), reactive rGE, and active rGE (Jaffee and Price 2007; Plomin et al. 1977). All of the aforementioned rGE could influence PRS-based risk estimates and may amplify the true genetic contribution to an individual’s traits.

We define G × E as interaction with a single genetic variant, and polygenic G × E as interactions involving multiple genetic variants. Empirical polygenic risk score by E interaction (PRS × E), a subset of polygenic G × E, specifically refers to interactions between a constructed PRS and E. In this article, we review methods for polygenic G × E interactions, and provide guidance on empirical PRS × E analyses, and the analysis of PRS estimation within family. We will explain different epidemiological study designs and their appropriate statistical methods for the analysis of PRS × E. These approaches provide advantages in practice that can jointly model multiple environmental variables, recover full cohort study power, and model or minimize rGE. Open databases of existing GWAS results and relevant metadata enable construction of PRS and investigation of how genetic and environmental factors jointly influence disease risk, advancing the translation of genetic discoveries into precision medicine applications.

## 2 An overview of polygenic G × E interactions

Statistical methods for polygenic G × E can be broadly categorized into four approaches (Durvasula and Price 2025; Miao et al. 2024). The first approach focuses on detecting the existence of interactions by testing for imperfect genetic correlation between environments (for categorical or binary E; for continuous E, quintiles can be used to categorize E) using bivariate linkage disequilibrium score regression (LDSC) (Bulik-Sullivan et al. 2015a,b) to estimate the genetic correlation between categories in E. This approach is locus-dependent and can be used to narrow the search space for E and outcome traits, thereby guiding GWIS analyses (Durvasula and Price 2025). The second approach aims to test for differential heritability (Durvasula and Price 2025; Yang et al. 2010) between environments. These approaches examine whether the genetic architecture of a trait varies systematically across environmental strata. A third approach is to quantify the extent to which phenotypic variation in a population is attributable to polygenic G × E interaction (Dahl et al. 2020; Ni et al. 2019; Robinson et al. 2017; Shin and Lee 2021). All these 3 approaches can be used to test for the existence of polygenic G × E. Approaches 1 and 3 can estimate contributions to trait variance but do not provide accurate estimates of interaction effect sizes. A recent method called PIGEON, has been developed to estimate covariant G × E through variance component analysis under a relaxed G-E independence assumption (i.e., zero correlation between additive SNP effects on E and SNP × E effects on outcome), aimed at quantifying and interpreting polygenic G × E effects (Miao et al. 2024, 2025). This approach obtains unbiased genome-wide polygenic G × E interaction effects with potential sample overlap between the original GWAS summary statistics and the GWIS summary statistics. However, all the above approaches only permit analysis of a single environmental exposure at a time, which restricts the assessment of correlated polygenic G × E effects. A fourth approach involves empirical PRS × E interaction analysis, where trait-associated variants identified in an independent GWAS are aggregated into a score and incorporated into a disease risk model. This approach has gained popularity among epidemiologists and clinicians because it can be easily implemented using available environmental and genotypic data for understanding the aetiology of diseases. For example, a recent study used this approach and identified evidence of interactions between PRS and tobacco-chewing habit for buccal mucosa cancer patients (Mhatre et al. 2025).

To interpret the results using the above approaches, Durvasula and Price (2025) classified polygenic G × E interactions into 3 scenarios: imperfect genetic correlation (locus-dependent G × E), varying genetic variance (non-locus-dependent G × E), and proportional amplification (non-locus-dependent G × E). We refer the latter two scenarios together as amplification. Imperfect genetic correlation, identified using the first approach, can be conceptualized as E modifying the effects of independent loci. Varying genetic variance amplification is indicated by significant PRS × E interaction effects (fourth approach) accompanied by differential heritability (second approach), and can be conceptualized as E modifying pathways that aggregate genetic effects across many loci, resulting in a scaling of genetic effects. Proportional amplification is indicated by significant PRS × E interaction effects without differential heritability, and can be conceptualized as E modifying both genetic and environmental contributions to disease risk. Notably, imperfect genetic correlation can coexist with either varying genetic variance or proportional amplification. Previous work has explicitly modeled mixtures of imperfect genetic correlations (scenario 1) and amplification (scenarios 2 and 3 combined) (Zhu et al. 2023). In the remainder of this review, we introduce several recent statistical advances developed for detecting amplification using empirical PRS × E interaction approaches.

Empirical PRS × E analysis offers several key advantages. First, it provides flexibility in modeling different types of outcomes and can incorporate multiple PRS × E interaction effects simultaneously, enabling investigation of independent interaction effects when correlations between E variables are suspected (Domingue et al. 2020; Young et al. 2016). A major challenge in studying environmental risk factors is that many of them are correlated with each other, creating uncertainty about whether an observed interaction between G and an environmental variable might be driven by its correlation with other environmental variables. Therefore, fitting multiple interaction effects jointly can help determine whether the environmental variables interact with G independently of their correlations with each other (Domingue et al. 2020; Young et al. 2016). Previous work (Young et al. 2016) has shown that the *FTO* × TV watching interaction on BMI appeared significant when tested alone, but disappeared in a joint model including physical activity, frequency of alcohol consumption, dietary variation, sleep duration, smoking, and socioeconomic status by *FTO* interactions. Second, given advances in GWAS, a PRS can capture the effects of many variants across the genome, approaching genome-wide coverage in the limit. Third, this approach provides effect size estimates, quantifying the magnitude of a clinical intervention or outcome. This is well-suited for translation to clinical and public health settings, where the goal is often to develop actionable risk prediction models or set thresholds for interventions. Fourth, this approach enables pathway-specific PRS × E analyses, where SNPs are curated into functionally related groups, allowing investigation of whether environmental factors modify genetic risk through specific biological mechanisms (Durvasula and Price 2025; James Gauderman et al. 2025; Westerman et al. 2026). It is important to ensure that there is no sample overlap between the GWAS used to derive PRS weights and the target population in which PRS × E interactions are assessed. When sample overlap is present, spurious PRS main effects and PRS × E interaction effects may arise, leading to biased estimates and inflated Type I error rates. Approaches for detecting and addressing sample overlap are discussed in Section 3.2. Another consideration is that when analyzing binary outcomes in empirical PRS × E studies, researchers should note that the results are obtained on the observed scale rather than the liability scale commonly assumed in genetic heritability frameworks (Durvasula and Price 2025).

## 3 Definition of PRS

We first assume that we can characterize genetic influence on a disease outcome as a PRS, defined as a weighted sum of risk alleles for many SNPs, i.e.,

(1) $${\text{PRS}}_{i}=\sum_{j}w_{j}G_{ij},$$

where $G_{ij}$ is the number of risk alleles (0, 1, 2) that individual i carries for SNP j, and $w_{j}$ is the corresponding weight. $w_{j}$ is usually a main effect estimate of the association between SNP j and the target disease outcome (e.g., log odds ratio) based on a large GWAS. We focus on complex diseases characterized by a genetic architecture in which the effects of variants are additive across many loci on the entire genome. To reduce the measurement error of PRS, family-based GWAS summary statistics can be used to construct PRS (Guan et al. 2025). Pre-computed PRS weights are publicly available through repositories such as the PGS Catalog (Lambert et al. 2021). Many methods have been developed to construct PRS from GWAS summary statistics or individual-level genotype and phenotype data, and comprehensive guides for PRS construction are available in the literature (Wan Choi et al. 2020).

Alternatively, researchers can construct PRS tailored for interaction detection. Interaction PRS (iPRS) are built from G × E effect estimates obtained through genome-wide interaction study (GWIS). Variance PRS (vPRS) are derived from genome-wide variance quantitative trait locus (vQTL) analyses and offer a key practical advantage: they can capture interactions without requiring measurement of specific environmental factors. But vPRS can only be applied to study continuous outcomes. A recent systematic comparison evaluated main-effect PRS, iPRS, and vPRS for detecting PRS × E interactions (Westerman et al. 2026). All PRSs were constructed using the clumping and thresholding (C+T) method. The study found that iPRS typically had the highest power for interaction detection, while main-effect PRS or vPRS performed better when the environmental exposure had specific distributional properties or measurement error.

Another group of work has jointly modeled the main genotype effect and G × E effect into the construction of PRS, assuming there are true underlying interaction effects in the prediction of a continuous outcome. Recent studies (Huang et al. 2026; Jayasinghe et al. 2024; Zhai et al. 2022) incorporated both genotype main effect and G × treatment interaction effect to predict treatment-related outcomes. A series of methods was developed, including a Bayesian shrinkage approach and penalized regression methods. Results showed substantial advantages over main-effect PRS in prediction accuracy, and the ability to capture treatment-specific predictive effects. The study further demonstrated theoretically that main-effect PRS have reduced heritability for the outcome trait when true underlying G × E interactions exist, unless a stringent condition of proportional main and interaction effects across all causal SNPs is met (Zhai et al. 2022).

An important practical limitation for this type of construction is the limited availability of GWIS summary statistics. Unlike GWAS summary statistics, which are increasingly accessible through public databases, GWIS results containing both main and interaction effect estimates remain scarce. This reflects the computational demands of interaction analyses and the smaller number of studies conducting GWIS. As a result, most researchers will continue to use main-effect PRS for PRS × E testing until GWIS summary statistics become more widely available through data repositories and consortium efforts. These methodology advances in PRS construction highlight the importance of selecting appropriate PRS methods based on the research question. The analytical frameworks presented in the following sections apply to pre-constructed PRSs, as discussed above.

### 3.1 PRS standardization

A recent practical guide for analyzing PRS recommends standardizing PRS distributions across the continuum of genetic ancestry using a PC-projection approach (Chen et al. 2024). Briefly, after calculating raw PRS in the target dataset, principal component analysis (PCA) is conducted using independent SNPs (e.g., $r^{2}<0.01$ within 5000 kb windows) in a reference dataset such as the 1000 Genomes Project (2015), which contains multi-ancestral groups and is publicly available. The SNP loadings derived from this PCA are then used to compute ancestry principal components (PCs), allowing genotypes from the target dataset to be projected onto the same PC space. The same raw PRS is calculated in the reference dataset using the same SNPs and weights as in the target dataset. For each individual k in the reference dataset, PRS is first regressed on the top 5 or 10 ancestry PCs for mean-adjustment:

$${\text{PRS}}_{k,\text{ref}}=a_{0}+a_{1}{\text{PC}}_{k1,\text{ref}}+\cdots +a_{5}{\text{PC}}_{k5,\text{ref}}+\epsilon_{k}^{\text{mean}}.$$

The residuals $r_{k}$ of the reference dataset PRS that account for mean differences in PRS distributions is

$$r_{k,\text{ref}}={\text{PRS}}_{k,\text{ref}}-{\overset{ˆ}{a}}_{0}-{\overset{ˆ}{a}}_{1}{\text{PC}}_{k1,\text{ref}}-\cdots -{\overset{ˆ}{a}}_{5}{\text{PC}}_{k5,\text{ref}}.$$

The variance adjustment is done on a second linear regression based on the square residuals $r_{k,\text{ref}}^{2}$ as a proxy for PRS variance, regressed on the same set of PCs in the reference dataset:

$$r_{k,\text{ref}}^{2}=b_{0}+b_{1}{\text{PC}}_{k1,\text{ref}}+\cdots +b_{5}{\text{PC}}_{k5,\text{ref}}+\epsilon_{k}^{\text{var}}.$$

The final standardized PRS for each individual i in the target dataset is

$${\text{PRS}}_{i}^{\text{standardized}}=\frac{{\text{PRS}}_{i}^{\text{raw}}-{\overset{ˆ}{a}}_{0}-{\overset{ˆ}{a}}_{1}{\text{PC}}_{i1,\text{target}}-\cdots -{\overset{ˆ}{a}}_{5}{\text{PC}}_{i5,\text{target}}}{\sqrt{{\overset{ˆ}{b}}_{0}+{\overset{ˆ}{b}}_{1}{\text{PC}}_{i1,\text{target}}+\cdots +{\overset{ˆ}{b}}_{5}{\text{PC}}_{i5,\text{target}}}}.$$

This standardization created a PRS distribution with mean 0 and variance 1 across ancestries, allowing genetic risk to be interpreted consistently regardless of ancestral background. This approach is especially important given the continuous spectrum of human ancestry (Ding et al. 2023). Note that for family-based study such as the case-parent trio design, the denominator can be the SD for residualized PRS with respect to each ancestry group g in the reference data, i.e., $\text{SD}\left(r_{\text{ref},g}\right)$, where the ancestry group of individual k is $A_{k}\in 𝒢$ and 𝒢={EUR,AFR,EAS,AMR,SAS}. So ${\text{PRS}}_{i}^{\text{standardized}}$ is calculated per ancestry group. The reason of using perancestry group standardization rather than using PC-projections in the denominator for each individual is to prevent the violation of Mendel’s law within families (Wang et al. 2026).

Two important considerations should be noted. First, standardization changes the interpretation of the PRS coefficient and interaction coefficient (per SD of ancestry-adjusted PRS), which can complicate cross-study comparisons. We therefore recommend that researchers document their complete standardization procedure, including the reference population used, to facilitate accurate interpretation and cross-study comparisons. Second, PC-projection mitigates ancestry-related mean and variance shifts in PRS distributions but does not remove confounding from population stratification or rGE. Additional strategies to account for these sources of bias are necessary when outcomes or environmental exposures are correlated with ancestry or geographic structure. We describe these strategies in the following sections.

### 3.2 Sample overlap between training GWAS and target data for PRS

A critical assumption for valid inference for PRS main effect and PRS × E interactions is that there is no sample overlap between the GWAS used to derive PRS weights and the target population in which PRS and PRS × E interactions are assessed. When sample overlap exists, spurious main and interaction effects related to the PRS can arise, leading to biased estimates and inflated Type I error rates. Methods for addressing sample overlap in PRS construction are an active area of methodological development (Choi et al. 2023; Ellis et al. 2024; Jeong et al. 2025). Bivariate LDSC (Bulik-Sullivan et al. 2015a,b) can be used to detect and quantify the degree of correlation due to sample overlap between the GWAS training dataset and the target dataset used for PRS evaluation. EraSOR (Choi et al. 2023) is a tool that implements this approach and generates decorrelated GWAS summary statistics for the base GWAS, which can then be used to construct PRS in the target sample. Note that this method requires both base and target cohorts with sample sizes greater than 1,000 for accurate adjustments, and is only consistently accurate when both cohorts have greater than 5,000 samples. Another study (Ellis et al. 2024) suggested that excluding overlapping or related individuals can perform as well as, or better than, statistical adjustment for overlap and relatedness. Thus, when overlapping samples are known, removal of these individuals represents an alternative to adjustment-based approaches such as EraSOR (Choi et al. 2023).

In particular, if a cohort is included in both the base GWAS and the target dataset, the ideal approach is to remove that cohort from the base GWAS and recompute the GWAS summary statistics. If individual-level genotype data from the full GWAS are not available, but the overlapping cohort GWAS results can be obtained, investigators can instead derive GWAS summary statistics for the base GWAS excluding the overlapping cohort using meta-inverse methods, and use these updated summary statistics to construct PRS. A recent tool has been developed to facilitate this approach (Jeong et al. 2025). Alternatively, when the overlapping cohort sample size is small, investigators may remove the overlapping individuals directly from the target dataset, provided that doing so does not substantially reduce statistical power.

The influence of sample overlap can be quantified by comparing PRS prediction performance using the original GWAS summary statistics with that using decorrelated summary statistics. A reduction in PRS association strength or predictive accuracy after decorrelation indicates that part of the original PRS performance was attributable to overlap-induced covariance rather than independent predictive signals.

Furthermore, PRS construction requires careful consideration of the match between the GWAS discovery population and the target population. Limited transferability across ancestries or populations can reduce predictive power and lead to attenuated estimates of both main genetic effects and PRS × E interactions (Kachuri et al. 2024).

## 4 Study designs and corresponding statistical methods for PRS × E interactions

In this section, we focus on study designs and statistical methods for empirical PRS × E interaction analysis, providing guidance for appropriate application in various epidemiological contexts. A workflow for the analysis is shown in Figure 1, and detailed study designs with corresponding methods are presented in Table 1.

### 4.1 Cohort study

To study the risk factors of a phenotype outcome, a rigorous prospective cohort design is the straightforward approach. Let $Y_{i}$ denote an outcome variable and ${\text{PRS}}_{i}$ denote the PRS value of an individual i. Let $E_{i}$ denote a vector of environmental variables of interest, and $C_{i}$ are a vector of additional covariates to be adjusted in the model. We consider a generalized linear model that incorporates PRS and environmental main effects, and PRS by environmental interaction terms as

(2) $$g\left\{E\left(Y_{i}|{\text{PRS}}_{i},E_{i},C_{i}\right)\right\}=\beta_{0}+\beta_{G}{\text{PRS}}_{i}+\beta_{E}^{T}E_{i}+\beta_{GE}^{T}E_{i}{\text{PRS}}_{i}+\beta_{C}^{T}C_{i},$$

where $E\left(Y_{i}|{\text{PRS}}_{i},E_{i},C_{i}\right)$ is the expected value of $Y_{i}$ conditional on ${\text{PRS}}_{i},E_{i}$, and $C_{i};g$ is the link function. The interaction term $\beta_{GE}$ provides evidence regarding the presence of PRS×E and quantifies the effect of interaction.

It is important to note that interaction effect estimates are scale-dependent. The interpretation and magnitude of interaction effects can differ substantially across scales. We use a binary disease outcome as an example here.

When Y is binary, a typical model used to test for interactions is on the multiplicative scale using the logit link function:

(3) $$\text{logit}\left\{\text{pr}\left(Y_{i}=1|{\text{PRS}}_{i},E_{i},C_{i}\right)\right\}=\beta_{0}+\beta_{G}{\text{PRS}}_{i}+\beta_{E}^{T}E_{i}+\beta_{GE}^{T}E_{i}{\text{PRS}}_{i}+\beta_{C}^{T}C_{i},$$

with $\beta_{0}$ being the log odds of the disease in a baseline category and $\beta =\left(\beta_{G},\beta_{E}^{T},\beta_{GE}^{T},\beta_{C}^{T}\right)$ being the log odds ratio parameters associated with different risk factors and their interactions.

Alternative scales, such as the additive scale for excess risk, can also be employed. This follows a linear model:

(4) $$\text{pr}\left(Y_{i}=1|{\text{PRS}}_{i},E_{i},C_{i}\right)=\alpha_{0}+\alpha_{G}{\text{PRS}}_{i}+\alpha_{E}^{T}E_{i}+\alpha_{GE}^{T}E_{i}{\text{PRS}}_{i}+\alpha_{C}^{T}C_{i}.$$

Furthermore, a log-linear model on the relative risk scale can also be employed:

(5) $$\text{log}\left\{\text{pr}\left(Y_{i}=1|{\text{PRS}}_{i},E_{i},C_{i}\right)\right\}=\gamma_{0}+\gamma_{G}{\text{PRS}}_{i}+\gamma_{E}^{T}E_{i}+\gamma_{GE}^{T}E_{i}{\text{PRS}}_{i}+\gamma_{C}^{T}C_{i}.$$

A practical limitation of linear and log-linear models is that they can produce predicted probabilities below 0 or above 1, leading to convergence failures in maximum likelihood estimation. Logistic regression overcomes this problem by using the logit link function, which ensures all predicted probabilities remain in the valid (0, 1) range. For this reason, current practice favors logistic regression for multiplicative interaction assessment. When disease prevalence is small at all levels of the environmental variable, the relative risk estimates will approximate odds ratio estimates for interaction effects (VanderWeele and Knol 2014).

Researchers have demonstrated that conceptual models of biological interaction correspond to interactions on the additive scale, not necessarily the multiplicative scale. Critically, for public health applications, such as deciding whether to target interventions to individuals with high genetic susceptibility, additive interactions and absolute risk differences are most informative (Basten et al. 2025; Han et al. 2012; Knol and VanderWeele 2012; Kowall et al. 2025; Weinberg 2012; Walter and Holford 1978). Recognition of this has led to recommendations that researchers routinely report interaction effects on both scales (VanderWeele and Knol 2014).

Under a single E variable, the relationship between the additive interaction, sometimes referred to as the relative excess risk due to interaction (RERI), and relative risk and odds ratios can be written as

$${\text{RERI}}_{RR}=\frac{\alpha_{GE}}{\alpha_{0}}=\text{exp}\left(\gamma_{GE}+\gamma_{E}+\gamma_{G}\right)-\text{exp}\left(\gamma_{G}\right)-\text{exp}\left(\gamma_{E}\right)+1\approx \text{exp}\left(\beta_{GE}+\beta_{E}+\beta_{G}\right)-\text{exp}\left(\beta_{G}\right)-\text{exp}\left(\beta_{E}\right)+1={\text{RERI}}_{OR}.$$

When the outcome is sufficiently rare, odds ratios approximate relative risks, allowing researchers to assess additive interactions using estimates from logistic regression model. However, the rare disease assumption (typically requiring prevalence <10%) must hold within every stratum of the environmental exposure E (VanderWeele and Knol 2014).

When Y is a quantitative trait and g is identity function, this follows a linear regression

(6) $$Y_{i}=\beta_{0}+\beta_{G}{\text{PRS}}_{i}+\beta_{E}^{T}E_{i}+\beta_{GE}^{T}E_{i}{\text{PRS}}_{i}+\beta_{C}^{T}C_{i}+\epsilon_{i},$$

where $\beta_{GE}$ is the additive interaction term.

Recent methods that leverage G × E effects in PRS construction often include both an additive PRS and an interaction-derived PRS as main effects in the model. The key term for statistical inference is the interaction-derived PRS × E interaction, which captures how the interaction-informed PRS varies across environmental exposures (Huang et al. 2026; Jayasinghe et al. 2024; Jayasinghe et al. 2025).

In cohort study, population stratification should always be addressed, covariates C can include top genetic PCs, geographic covariates, self-reported race, and study centers. Previous literature has reviewed these approaches (Price et al. 2010; Lin et al. 2023), though recent work has suggested that over-adjustment for PCs could introduce collider bias in admixed populations (Grinde et al. 2024). Note that in population-based studies such as cohort studies, estimated main effects remain susceptible to biases from assortative mating or indirect parental effects. Therefore, when possible, comparing results with those from family-based studies is advisable as a sensitivity analysis to assess potential bias.

### 4.2 Case-control study

When the disease is rare, a case-control design is more desirable given the advantages of low-cost and time-efficient of collecting covariates (Lewallen and Paul 1998). A case-control study follows a retrospective sampling scheme, where individuals are selected based on their disease status, and genotypic or phenotypic information is ascertained in both cases and controls (Piegorsch et al. 1994). The classical results of Prentice and Pyke (1979) established the invariance property of the odds ratio in general logistic regression for case-control sampling designs, making this computationally simple method a standard approach for analyzing risk factors in such studies. With the ease of arbitrary marginal distributions of the risk factors, logistic regression has also been widely used to study the G × E or PRS × E interactions in case-control studies. G × E interaction problems come with a special characteristic that a person’s genetic factors can be independent with many subsequent environmental exposures. However, this characteristic cannot be leveraged by logistic regression that allows arbitrary marginal covariate distributions in the model. There exists, however, opportunities for more efficient inference in case-control studies exploiting a retrospective likelihood of the data. In particular, for the analysis of interactions using a SNP, a variety of methods (Chen et al. 2009; Chatterjee and Carroll 2005; Hsu et al. 2012; James Gauderman et al. 2017; Murcray et al. 2009; Umbach and Weinberg 1997) have been developed to explore an assumption of G-E independence in the underlying population and to enhance the efficiency of inference on interaction odd-ratio parameters. Conversely, it has been shown that rGE can be estimated efficiently by pooling cases and controls from a case-control study in the absence of G × E interactions (Li et al. 2010). In the context of PRS × E interactions, one general semiparametric method (Stalder et al. 2017) assumes arbitrary parametric modeling of multiple genetic variants by evaluating the expectation terms empirically using retrospective likelihoods. This method shows great flexibility and efficiency gain while requires strict G-E independence assumption. When the independence assumption is violated, there could be spurious PRS × E results. A recent method has been proposed that leverages the G-E independence assumption to enhance the efficiency of PRS × E interaction parameters, while jointly modeling potential rGE within the same framework for bias-variance trade-off in a case-control design (Wang et al. 2024).

We briefly introduce this method below (see Figure 2). Let Y be the disease outcome, E be a vector of environmental variables of interest. Let S be a vector of factors that may influence the distribution of the PRS in the underlying population. This includes endogenous environmental factors that have heritable components and may be genetically correlated with the disease of interest, as well as social determinants of health and population stratification factors. For example, S may include genetic PCs, self-reported race, study centers, Townsend deprivation index, or geographical regions. Assume pr(PRS∣E,S)=pr(PRS∣S), i.e., PRS is independent of E conditional on S. Define

(7) $$\text{PRS}|S\sim N\left(\eta_{s}^{T}S,\sigma_{s^{c}}^{2}\right),$$

where in the mean component, $\eta_{s}$ denotes a set of unknown parameters characterizing the linear correlation between PRS and S, and the variance of the normal distribution is allowed to vary by the levels of a categorical stratification variable $S^{c}$, assumed to be a coarsened version of S. As PRS is typically defined by a combination of many genetic variants, the normality approximation is typically accurate due to the central limit theorem. Further, readers have the flexibility to specify the mean model and embed any assumption of G-E independence or dependence by the exclusion or inclusion of the corresponding E/S variables. Moreover, this model allows for heteroscedasticity in the distribution of PRS, but here instead of specifying a regression model, the variance parameters freely vary according to a user-specified categorical variable. In general, S can include continuous and categorical variables, such as the top 10 PCs, self-reported race/ethnic groups, and geographic regions; and $S^{c}$ can be constructed by one or a combination of multiple categorical variables such as race/ethnic groups and geographic regions.

Let X=(E,S), and the disease model of interest is now

(8) $$\text{logit}\left\{\text{pr}\left(Y_{i}=1|{\text{PRS}}_{i},X_{i}\right)\right\}=\beta_{0}+\beta_{G}{\text{PRS}}_{i}+\beta_{X}^{T}X_{i}+\beta_{GX}^{T}X_{i}^{I}{\text{PRS}}_{i},$$

where $\beta ={\left(\beta_{G},\beta_{X}^{T},\beta_{GX}^{T}\right)}^{T}$, with $X^{I}\subseteq X$ involves the environmental and stratification variables for which interactions are to be incorporated into their relationship to the PRS variable.

Under a case-control design, $N_{1}$ cases and $N_{0}$ controls are sampled from an underlying population for which the above models hold. The retrospective likelihood for case-control data takes the form $L^{R}=\prod_{i=1}^{N_{0}+N_{1}}\text{pr}\left({\text{PRS}}_{i},E_{i},S_{i}|Y_{i}\right)$. Note that the joint distribution function for (E,S) is completely unspecified. The maximum likelihood estimates for the parameters of interest, namely $\beta ,\eta_{s}$, and $\sigma_{s^{c}}^{2}$ can be obtained using the profilelikelihood techniques, which removes high-dimensional nuisance parameters associated with the distribution of pr(E,S), can be approximated under a rare disease assumption as

$$L^{R}=\prod_{i=1}^{N_{0}+N_{1}}\frac{f_{\text{PRS}|S}\left({\text{PRS}}_{i}|S_{i}\right)\text{exp}\left[Y_{i}\left\{\kappa +\beta_{G}{\text{PRS}}_{i}+\beta_{X}^{T}X_{i}+\beta_{GX}^{T}{X^{I}}_{i}{\text{PRS}}_{i}\right\}\right]}{1+\text{exp}\left\{\kappa +\frac{1}{2}\sigma_{s^{c}}^{2}{\left(\beta_{G}+\beta_{GX}^{T}X_{i}^{I}\right)}^{2}+\eta_{s}^{T}S_{i}\left(\beta_{G}+\beta_{GX}^{T}X_{i}^{I}\right)+\beta_{X}^{T}X_{i}\right\}},$$

with a nuisance intercept $\kappa =\beta_{0}+\text{log}\left(N_{1}/N_{0}\right)-\text{log}\left(\pi_{1}/\pi_{0}\right)$. The intercept $\beta_{0}$ in the disease model (8) can be estimated with the above likelihood only if the disease prevalence $\pi_{1}=\text{pr}(Y=1)$ in the underlying population is known *a priori*. In a situation of frequency-matched case-control design, this method can be easily modified by defining intercept terms $\kappa_{w_{m}}$ for each of the M strata in the matching variables $W=w_{m}(m=1,\cdots ,M)$.

### 4.3 Case-only study

When sampling both cases and controls from the population is challenging, and the gene-environment interaction is the parameter of primary interest, it is useful to employ a type of study design that samples cases only (Piegorsch et al. 1994; Schmidt and Schaid 1999; Tchetgen and Robins 2010; VanderWeele et al. 2010). Recent work in polygenic gene-environment interactions include a case-only method (Allison et al. 2019) which is derived upon a log-linear disease model with the assumptions of rare disease and gene-environment independence. This method shows great efficiency gain in the multiplicative interaction parameter estimation based on cases alone than a logistic regression model in case-control study. The case-only method allows the estimation of main effects of PRS and PRS × E interaction parameters but not the main effect of environmental variables given that there is missing information of the controls. A log-linear model is used to model disease risk as in formula (5) for a E variable. Note that when the disease is rare at all levels of E, the relative risk estimates will approximate odds ratio estimates.

Under PRS-E independence assumption, define $\text{PRS}\sim N\left(\mu ,\sigma^{2}\right)$, it can be shown that

(9) $$\text{PRS}|E,Y=1\sim N\left(\mu +\sigma^{2}\left(\beta_{G}+\beta_{GE}E\right),\sigma^{2}\right).$$

Therefore, it follows that fitting a simple linear regression of PRS on E to a sample of cases can provide estimates of ${\overset{ˆ}{\beta }}_{GE}$ and main effect ${\overset{ˆ}{\beta }}_{G}$. The estimated mean and variance of PRS itself can be obtained from an external dataset with a similar context or a sample of controls when the disease is rare. One such publicly available resource is the 1000 Genomes Project (2015). This study design, however, is highly sensitive to the assumption of independence between G and E. Even when G and E are mildly correlated, the method may yield false positive multiplicative statistical interactions. Albert et al. (2001) suggested testing for associations between G and E in controls before conducting the case-only study. However, such an approach may still lead to an inflated type I error rate when the association test in control samples has limited power. Furthermore, VanderWeele et al. (2010) (VanderWeele et al. 2010) argued that case-only design results may not reflect mechanistic multiplicative interactions except under specific conditions. Therefore, this study design is best used as a screening approach for PRS × E interactions, and further sensitivity analyses are warranted in data applications (Jaffee and Price 2007; VanderWeele et al. 2010).

### 4.4 Family-based study

Previous methodological work in genetic studies of unrelated individuals has addressed population stratification through genomic control (Devlin and Roeder 1999), PCA (Price et al. 2006), linear mixed models (Loh et al. 2015), and linkage disequilibrium score regression (Bulik-Sullivan et al. 2015), which reduce potential bias across populations and geographical regions. In previous sections, we primarily used PCA to correct for potential biases induced by population stratification. However, rGE can arise from sources beyond population stratification that cannot be easily addressed in population-based studies. Notably, recent work has analyzed PRS using non-transmitted parental alleles, demonstrating that parental genotypes can affect children’s phenotypes through the family environment they create – an indirect genetic effect (Kong et al. 2018). In population-based GWAS, the main effect estimate captures both direct and indirect genetic effects. This conflation leads to overestimated effect sizes when parental indirect effects are not explicitly modeled. Additionally, assortative mating effects are difficult to address in standard designs. These confounding effects can be mitigated through an entirely different class of study designs: family-based designs, where genetic studies are conducted within families, naturally controlling for shared familial environment. In twin studies, PRS analyses have employed mixed-effect modeling approaches (Selzam et al. 2019). For case-parent trio designs, the polygenic transmission disequilibrium test (pTDT) (Weiner et al. 2017) was developed to detect whether PRS affects disease risk in probands. These methods are not developed for estimating PRS × E interactions. One study employed conditional logistic regression in family tetrads using data from the Sister Study and Two Sister Study cohorts, prospective family-based studies that enrolled unaffected women with sisters diagnosed with breast cancer and subsequently collected genotype data from cases and their parents (Shi et al. 2020). This tetrad design enabled robust estimation of PRS × E interactions by leveraging within-sibling comparisons of environmental exposures, thereby reducing potential confounding bias from population stratification in E. Another recent approach called PGS-TRI, used case-parent trio designs to estimate the direct and indirect effects of PRS as well as PRS × E interactions (Wang et al. 2026). We briefly introduce this method below.

Case–parent trio studies recruit affected individuals (probands) from a population and genotype them alongside both biological parents. The elegance of this design lies in its use of Mendelian inheritance: each parent carries two alleles at every locus but transmits only one to their child. The untransmitted alleles thus provide well-matched controls, eliminating the need for an independent control cohort (Duncan 2010; Irfanulla Khan and Prashanth 2020; Zhang et al. 2021). The family-based studies generally have lower statistical power compared to population-based studies due to genotype overmatching (Duncan 2010). However, they can be more powerful for G × E interaction testing, as within-family matching creates more genotype-concordant, exposure-discordant pairs than population sampling, especially when exposures aggregate in families (Duncan 2010; Witte et al. 1999). For direct genetic effects, case-parent trio design is immune to population-stratification bias, however, for G × E interaction, a weak assumption that the proband’s environmental exposure and genotype are independent conditional on parental genotypes is required (Duncan 2010; Wang et al. 2026).

Let ${\text{PRS}}_{C}$ denote the PRS value for the child, ${\text{PRS}}_{M}$ the PRS for mother and ${\text{PRS}}_{F}$ the PRS for father. Let $Y_{C}$ denote the binary disease status for the child. We assume i=1,⋯,N families have been sampled following the case-parent trio design. We also assume a set of environmental exposures, denoted by $E_{ic}$, has been ascertained for each child across the different families. We assume prospective disease risk of the child in the population follows a log-linear model:

$$\text{pr}\left(Y_{iC}|{\text{PRS}}_{iC},{\text{PRS}}_{iF},{\text{PRS}}_{iM},E_{iC}\right)=\text{exp}\left(\alpha_{i}+\beta_{G}{\text{PRS}}_{iC}+\beta_{F}{\text{PRS}}_{iF}+\beta_{M}{\text{PRS}}_{iM}+\beta_{GE}^{T}E_{iC}{\text{PRS}}_{iC}+\beta_{E}^{T}E_{iC}\right),$$

here, $\beta_{G}$ is the direct genetic effect, $\beta_{M}$ and $\beta_{F}$ are the parental indirect genetic effects on children’s disease risk. $\alpha_{i}$ is family-specific intercept terms without any further assumption about their distribution, thereby allowing disease risks to vary arbitrarily across families. $\beta_{GE}^{T}$ are a vector of PRS × E interaction effects. $E_{ic}$ is a vector of environmental variables of the child in family i. These variables can be defined arbitrarily without distributional assumptions; for example, they may include the child’s exposure to maternal smoking during pregnancy. Importantly, because this model makes comparisons within families, it inherently controls for confounding factors that would typically need to be explicitly included in population-based models. Mathematically, the main environmental effects $\beta_{E}^{T}$ cancel out in the derivation, analogous to conditional logistic regression.

Assume that the distribution of ${\left({\text{PRS}}_{iC},{\text{PRS}}_{iM},{\text{PRS}}_{iF}\right)}^{T}$ in each family i follows

(10) $$\left(\begin{array}{l} {\text{PRS}}_{iC}\\ {\text{PRS}}_{iM}\\ {\text{PRS}}_{iF} \end{array}\right)\sim 𝒩\left(\mu_{i}1_{3},\sigma_{i}^{2}\left(\begin{array}{ccc} 1 & \frac{1}{2} & \frac{1}{2}\\ \frac{1}{2} & 1 & 0\\ \frac{1}{2} & 0 & 1 \end{array}\right)\right),$$

where $\mu_{i}$ and $\sigma_{i}$ are the mean and standard deviation for each family i without imposing any assumptions regarding their distributions.

The retrospective likelihood for the case-parent trio data in each family i can be decomposed into an offspring’s $\left(L_{iC}\right)$ and a parents’ $\left(L_{iP}\right)$ component as

$$L_{i}=\text{pr}\left({\text{PRS}}_{iC},{\text{PRS}}_{iM},{\text{PRS}}_{iF}|E_{iC},Y_{iC}=1\right)\;=\text{pr}\left({\text{PRS}}_{iC}|{\text{PRS}}_{iM},{\text{PRS}}_{iF},E_{iC},Y_{iC}=1\right)\times \text{pr}\left({\text{PRS}}_{iM},{\text{PRS}}_{iF}|E_{iC},Y_{iC}=1\right)\;=L_{iC}\times L_{iP}.$$

Under the above framework, the final estimator can be derived in an analytic form as a solution to a weighted least-square problem as

(11) $$\overset{ˆ}{\beta }={\left(E^{T}WE\right)}^{-1}E^{T}Z,$$

where $\overset{ˆ}{\beta }={\left({\overset{ˆ}{\beta }}_{G},{\overset{ˆ}{\beta }}_{GE}^{T}\right)}^{T},E_{N\times K}={\left(E_{1}^{T},\cdots ,E_{N}^{T}\right)}^{T},E_{i}={\left(1,E_{iC}^{T}\right)}^{T},W=diag\left({\overset{ˆ}{\sigma }}_{1}^{2},\cdots ,{\overset{ˆ}{\sigma }}_{N}^{2}\right)$, and $Z=\left(2\left({\text{PRS}}_{1C}-\right.\right.{\left.\left.\mu_{1C}\right),\cdots ,2\left({\text{PRS}}_{NC}-\mu_{NC}\right)\right)}^{T}$.

The gene–environment interactions in equation (11) has a natural interpretation. The quantity $E^{T}Z$ represents the sample covariance between transmission disequilibrium statistic and environmental variables across families. If no gene–environment interaction exists, we expect Mendelian transmission to be independent of children’s environmental exposures within families. This equation shows that departures from this null expectation in case–parent trio data provide evidence of gene–environment interaction on the multiplicative scale. Note that this transmission-based approach is generally applicable to family-level or parent-specific environmental factors (e.g., maternal smoking status during pregnancy) and to children’s own characteristics such as sex and genetic PCs. However, it is not appropriate when the environmental variable is a child’s trait suspected of correlation with the child’s target outcome through genetic pleiotropy, as such correlation between E and PRS cannot be fully accounted for by conditioning on parental PRS (Wang et al. 2026).

## 5 Discussion

The questions in gene-environment interactions and correlations for diseases have always been entangled with each other. In this review, we have outlined methods and study designs for analyzing PRS × E interactions and addressing gene-environment correlations, with particular emphasis on empirical approaches that can be readily implemented by epidemiologists and clinicians. Several critical considerations emerge from our review. First, the choice of study design – whether cohort-based, case-control, case-only, or family-based – has important implications for the validity and interpretability of PRS × E estimates. Each design offers distinct advantages for addressing specific sources of bias, particularly those arising from rGE. Second, the ability to jointly model multiple environmental exposures is essential given the pervasive correlations among behavioral, sociocultural, and physical environmental factors. Failing to account for these correlations can lead to overestimation of interaction effects, as an observed PRS × E association may reflect confounding with correlated but unmeasured exposures rather than an independent effect. We recommend that investigators pre-specify environmental exposures based on prior literature and biological mechanisms, report both one-at-a-time and joint models when possible, and apply appropriate corrections for multiple testing when evaluating multiple PRS × E hypotheses. Today we have large resources and biobanks that simultaneously measure genetic, lifestyle and phenotypic information to enable us to do such type of joint modeling. This is an advantage over previous analyses that have tested only one interaction at a time (Domingue et al. 2020; Young et al. 2016). Third, within-family designs provide a powerful framework for disentangling direct genetic effects from indirect genetic effects mediated through the family environment, offering more robust estimates of PRS and PRS × E interactions in the presence of rGE.

Gene-environment correlation poses a fundamental challenge to the interpretation of PRS and PRS × E interactions. As we have discussed, rGE can arise through multiple mechanisms – including pleiotropy, population stratification, geographic variation, and within-family processes – each with different implications for causal inference and risk prediction. Methods that explicitly model or control for rGE, such as within-family analyses and approaches that leverage multiple environmental variables, are essential for obtaining unbiased estimates of how genetic risk is modified by environmental exposures. Researchers must carefully consider the potential sources of rGE in their study populations and select analytical approaches accordingly.

Despite methodological advances, several challenges remain. Large-scale studies examining PRS × E interactions across diverse populations are still scarce, limiting our understanding of how gene-environment interplay varies across ancestries and environmental contexts. An observed interaction between PRS and environment may indicate that the influence of genetic factors on the outcome is larger in some environments than others, that PRS accuracy varies across environments due to differences in genetic ancestry or population structure relative to the discovery GWAS, or a combination of the two. Distinguishing among these explanations for PRS research requires careful consideration of study design and population characteristics. The limited transferability of PRS across populations of different ancestries further complicates the application of PRS × E findings to diverse populations. Future research should establish standardized reporting protocols to facilitate reproducibility and meta-analysis.

Additionally, as sequencing technologies advance, there is a growing need for frameworks that incorporate the full spectrum of genetic variation – including rare variants and structural variants – into PRS × E analyses. Pathway-specific and functionally curated PRS offer promising avenues for elucidating the biological mechanisms underlying gene-environment interactions and may provide more interpretable and actionable insights for precision medicine.

As GWAS continue to grow in size and diversity, and as PRS become increasingly integrated into healthcare systems, a sophisticated understanding of gene-environment interplay will be essential for realizing the promise of precision medicine. By carefully considering study design, appropriately modeling multiple environmental factors, and addressing gene-environment correlation, researchers can generate robust insights into how genetic and environmental factors jointly shape disease risk – ultimately advancing both scientific understanding and public health impact.

## Figures and Tables

**Figure 1:**
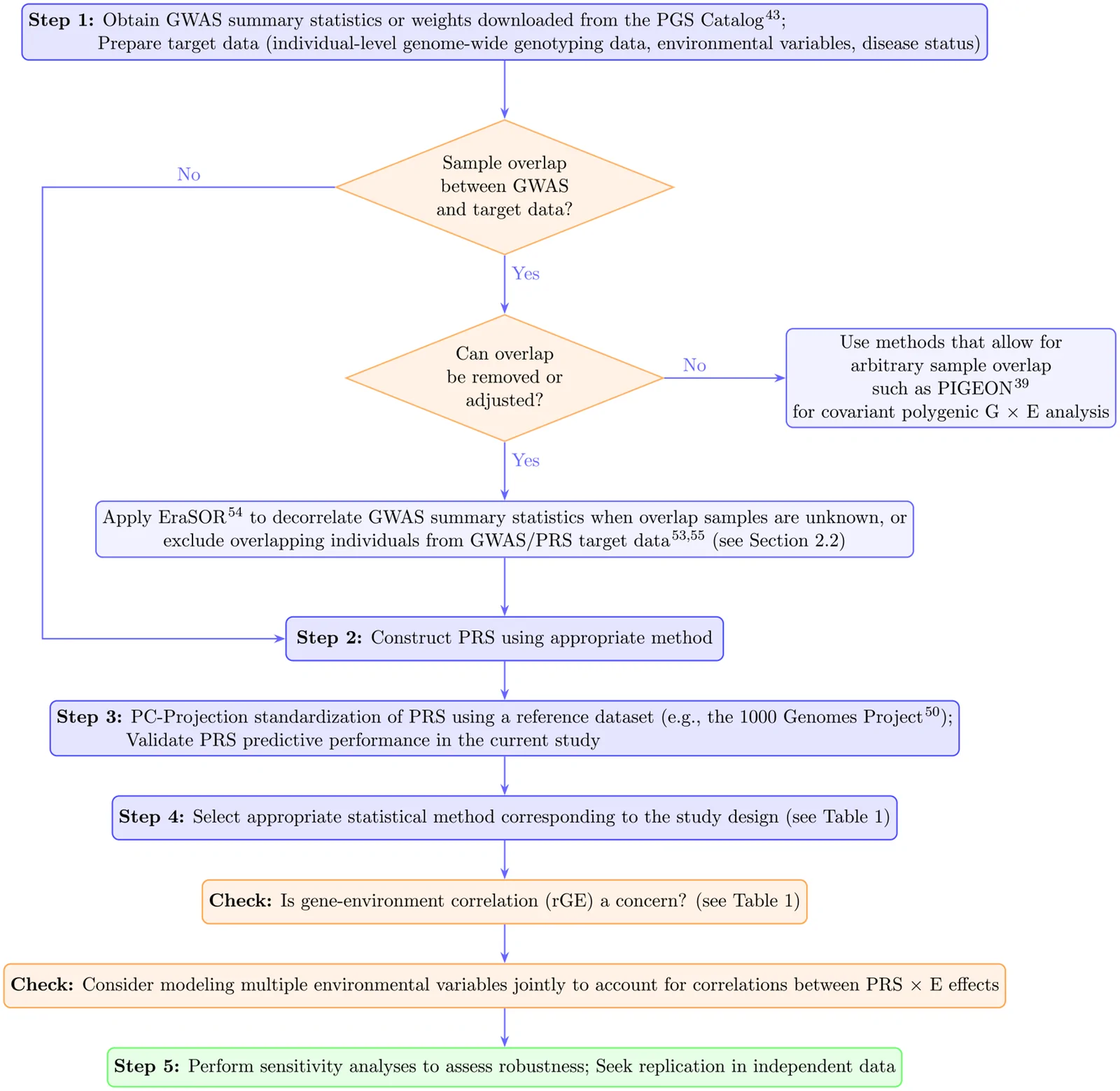
Workflow for performing PRS × E interaction analysis, including handling of sample overlap and alternative methods.

**Figure 2:**
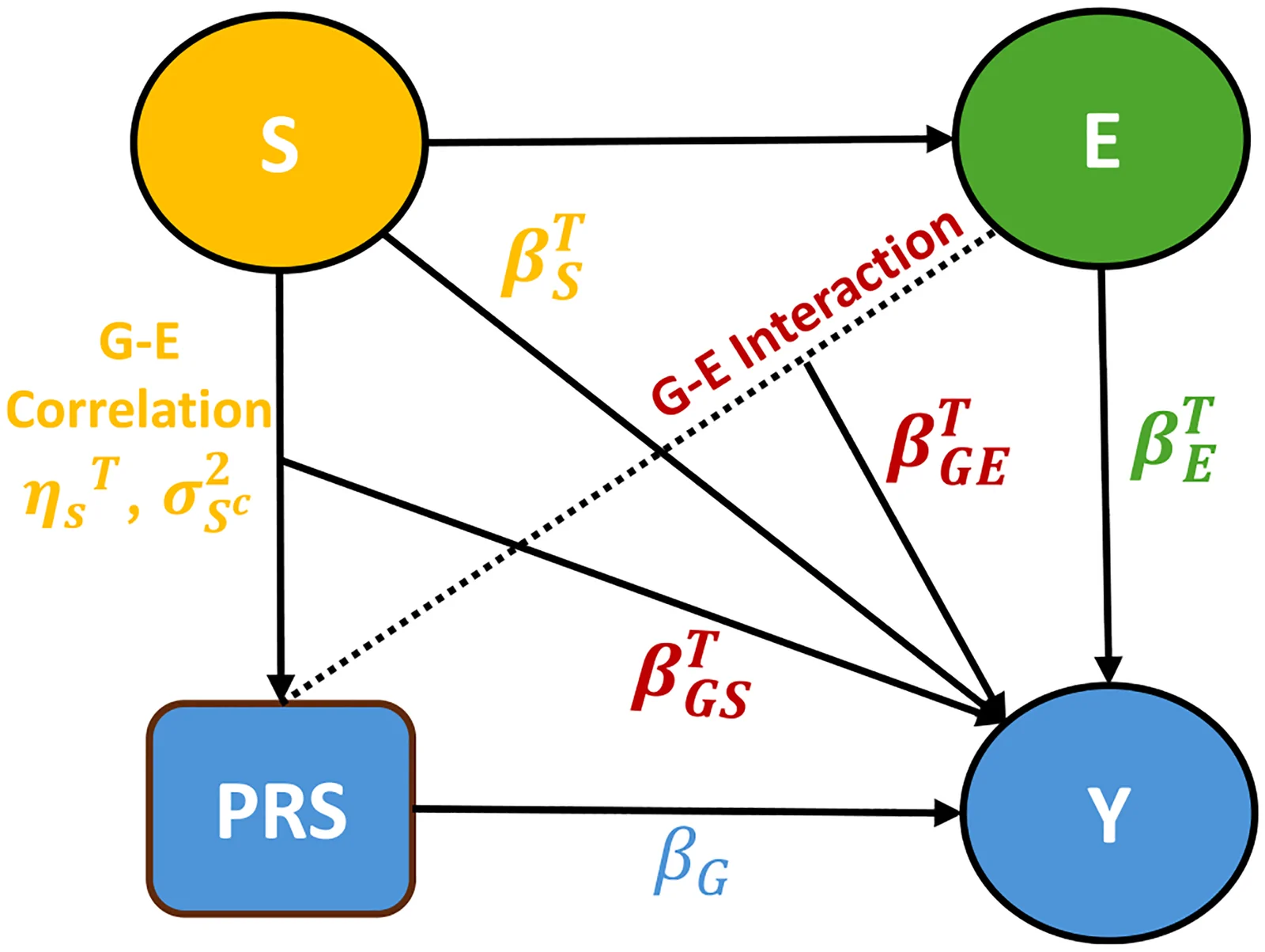
A figure illustration of G-E interactions and correlations with respect to a disease outcome, and the model setup of the retrospective likelihood method (Wang et al. 2024) for efficiently estimating PRS × E and rGE in a case-control study. Y, outcome of interest; E, a vector of environmental factors; S, a vector of stratification factors; PRS is independent of E conditional on S. $\beta_{G}$, main effect of PRS; $\beta_{E}^{T}$ and ${\beta_{s}}^{T}$, vectors of main effects of E and $S;\beta_{GE}^{T}$, a vector of interaction parameters for PRS; ${\beta_{GS}}^{T}$, a vector of interaction parameters for PRS; ${\eta_{s}}^{T}$, a vector of rGE parameters; $\sigma_{s^{c}}^{2}$, variance for PRS∣S corresponding to a coarsened version of S.

**Table 1:** Study designs for PRS × E interaction analysis and corresponding statistical methods.

Study design	Statistical methods	Outcome type	Advantages	rGE control	Software
Prospective cohort	Generalized linear regression with PRS × E	Continuous, binary^a^	Absolute risk estimation; gold standard for risk prediction	Population stratification control needed^b^	https://www.cog-genomics.org/plink/2.0/assoc#glm
	GxEprs (Jayasinghe et al. 2024)	Continuous, binary	Enhance PRS prediction accuracy by utilizing G × E effects^c^	Population stratification control needed^b^	https://github.com/DoviniJ/GxEprs
	GEiPRS (Huang et al. 2026)	Continuous	Enhance PRS prediction accuracy by utilizing G × E effects^c^	Population stratification control needed^b^	https://github.com/linnabrown/geiprs
Case-control	Logistic regression with PRS × E	Binary	Cost and time efficient for rare diseases	Population stratification control needed^b^	https://www.cog-genomics.org/plink/2.0/assoc#glm
	Semiparametric retrospective likelihood approach (Stalder et al. 2017)	Binary	Cost and time efficient for rare diseases; more efficient than logistic regression; arbitrary distribution of PRS	Strict G-E independence assumption required	https://github.com/alexasher/caseControlGE
	Joint modeling retrospective likelihood approach (Wang et al. 2024)	Binary	Cost and time efficient for rare diseases; more efficient than logistic regression	Jointly model interaction and rGE	https://github.com/ziqiaow/RetroGE
Case-only	Regression of PRS on E (Allison et al. 2019)	Binary	Most efficient; no controls needed; can be used as a quick screening step	Strict G-E independence assumption required	https://github.com/ziqiaow/PGS.TRI/blob/main/R/case-only.R
Within-family	Conditional logistic regression (siblings, tetrad) (Shi et al. 2020)	Binary	Controls shared environment, unbiased interaction effects	Yes	https://rdrr.io/bioc/CGEN/man/snp.matched.html
	PGS-TRI (case parent trios) (Wang et al. 2026)	Binary	Can separate direct and indirect genetic effects; controls stratification and assortative mating	Yes^d^	https://github.com/ziqiaow/PGS.TRI

**rGE** = gene-environment correlation; **PC** = principal components.

a Implemented via appropriate exponential family distributions and link functions in the generalized linear (mixed) model framework.

b Adjustment for covariates such as top 10 genetic PCs, geographic covariates, self-reported race, and study centers. See Section 4.1. for more details.

c Requires large training sample size to obtain G × E effects.

d PRS × E requires weak independence assumption within each family. Population-based designs (cohort, case-control) should adjust for population stratification. This study design does not prevent biases from assortative mating or genetic indirect effects. Family-based designs provide control for population stratification and assortative mating, case-parent trios can further separate genetic direct and indirect effects. Cases should be incident, not prevalent, occurring after G and E measurement. An overview of other approaches for polygenic G × E analyses (including imperfect genetic correlations, differential heritability, and proportion of variation) is in Section 2.

## Abbreviations

GWAS: genome-wide association studies
PRS: polygenic risk scores
G-E: gene-environment
PRS × E: PRS and environment interaction
rGE: gene-environment correlation
SNP: single-nucleotide polymorphism
PCA: principal component analysis
